# Impact of River Water and Bottom Sediment Pollution on Accumulation of Metal(loid)s and Arsenic Species in the Coastal Plants *Stuckenia pectinata L*., *Galium aparine L*., and *Urtica dioica L*.: A Chemometric and Environmental Study

**DOI:** 10.1007/s00244-020-00727-w

**Published:** 2020-04-13

**Authors:** Magdalena Jabłońska-Czapla, Piotr Zerzucha, Katarzyna Grygoyć

**Affiliations:** 1 Institute of Environmental Engineering of the Polish Academy of Sciences, 34 M. Skłodowska-Curie Street, 41-819 Zabrze, Poland; 2 Faculty of Philosophy, The Pontifical University of John Paul II, 9 Kanonicza Street, 31-002 Kraków, Poland

## Abstract

The role of water and bottom sediment pollution of a river subjected to a strong industrial anthropo-pressure in coastal plants was investigated. The work presented the influence of polluted environment on accumulation of metal(loid)s (including arsenic and its species) in *Stuckenia pectinata L.*, *Galium aparine L*., and *Urtica dioica L.* The study provided important information on the contents of organic and inorganic arsenic species in selected plants and their response to heavy metal and arsenic contamination. The As(III), As(V), AB (arsenobetaine), MMA (monomethylarsonic acid), and DMA (dimethylarsinic acid) ions were successfully separated on the Hamilton PRP-X100 column with high-performance liquid chromatography-inductively coupled plasma-mass spectrometry (HPLC-ICP-MS) techniques. The Pollution Load Index and geo-accumulation Index (*I*_geo_) values clearly indicate significant pollution of the examined ecosystem with heavy metals. The chemometric analysis with the concepts of (Dis)similarity Analysis, Cluster Analysis, and Principal Component Analysis helped to visualize the variability of the As species concentrations and to analyse correlations between sampling point locations and analyte contents.

Environmental pollution in Upper Silesia region has been the highest in Poland for a decades. This has been due to the intense and unsustainable process of industrialization in the past two centuries. The mining and metallurgical industry predominates in this region.

As a result of many years of neglect, the Upper Silesian Rivers are still a potential source of pollution for other Polish regions. Silesian rivers are one of the most polluted rivers of Poland. Because they are tributaries of the main rivers (Vistula and Oder), they spread pollution throughout the country (Ciszewski and Czajka 2009). Rivers are exposed to pollution in the form of sewage, industrial and municipal water discharges, and atmospheric precipitation, as well as dust from the air (Morillo et al. 2002). Metals and metalloids are the leader in this context (Baldantoni et al. 2003).

The previous research has shown high levels of pollution of the Bytomka River, especially due to the high content of metals and metalloids in water, as well as in bottom sediments (Jabłońska-Czapla et al. 2014). Major sources of this type of pollution are old and inactive landfills of mining waste, which are currently abundant in Upper Silesia. In the Bytomka River concentrations of heavy metals, especially zinc, lead, and cadmium often have exceeded the values adopted for the current classification of surface water purity (Nocoń 2006). Furthermore, it has been shown that not only the total content of the elements but also the species in which they occur has a crucial effect on living organisms. The elements that arouse the greatest interest of toxicologists and analysts are antimony, arsenic, and chromium (Jabłońska-Czapla 2015).

Numerous studies have shown that ions, such as As(III) and Cr(VI), can cause many serious diseases. Therefore, genotoxicity studies and studies on the metabolism of arsenic and chromium ions in organisms inhabiting the ecosystems of rivers subjected to strong anthropogenic pressure are extremely important (Komorowicz and Barałkiewicz 2011; Selene et al. 2003; Templeton and Fujishiro 2017). Arsenic is a toxic metalloid that is common in various biological systems and in the environment. The contact with arsenic can cause various health effects, such as dermatological, inhalatory, cardiologic, genetic, genotoxic, or mutagenic lesions (Chou and De Rosa 2003). As toxicity depends on its chemical form: organic or inorganic. Studies have shown that its inorganic forms are 100 times more toxic that the organic ones. As(III) compounds show greater toxicity than As(V) compounds, which in turn are more toxic than organic forms. Arsenic compounds also have a latent carcinogenic and teratogenic effect (Kabata-Pendias and Pendias 1999; Majtkowski et al. 2011).

A study of analytes at low concentration levels, particularly in complex matrix samples, requires complex and sophisticated analytical methods and techniques. The latest trends in this field are concerned with the so-called hyphenated methods (such as high-performance liquid chromatography-inductively coupled plasma-mass spectrometry [HPLC-ICP-MS]), in which the separation and detection methods are coupled (Michalski et al. 2011, 2013a, b).

Under certain conditions (e.g., extreme rainfall resulting in flash floods), the contaminants accumulated in sediments may be remobilized into overlying waters. River sediments have a strong adsorption capacity for pollutants and thus many of the water column characteristics are inherited by sediments (Pekey et al. 2004). Sediments can be sensitive indicators for monitoring contaminants in aquatic environments (Tomlinson et al. 1980). One of the parameters for the assessment of the heavy metal contamination level is the Pollution Load Index (PLI) (Chakravarty and Patgiri 2009; Liu et al. 2013), which allows for an in-depth characterization of the degree of heavy metal pollution of the ecosystem.

Plants growing on the riverbanks are exposed to contaminants present in water and bottom sediment, becoming a specific indicator of ecosystem pollution. Plants, such as *Hypericum performatum L.*, *Mentha longifolia L.*, *Urtica dioica L*. or *Solidago virgaurea L.*, have developed specific mechanisms that allow them to survive in a heavily contaminated ecosystem (Łaszewska et al. 2007). Arsenic compounds are relatively easily extracted from the substrate, and the arsenic content in plants is proportional to its content in the soil. This means a passive mechanism of arsenic collection. Some plants have the ability to accumulate arsenic, usually in leaves and roots (Marchand et al. 2014). Arsenic uptake takes place not only from the ground through the root system but also from the air (dust, precipitation) through the leaves. Plants from areas not directly exposed to arsenic contamination contain from 3 to 1500 ng/mL, depending on the plant species and the plant organs (Kabata-Pendias and Pendias 1999). In the case of soil (or air) contamination with arsenic compounds, its content may increase to several thousand ppm (Niedzielski et al. 2000). Some plants have a greater tendency to accumulate pollutants from the environment, and they can be used to remove contamination in a phytoremediation process.

The plants with such properties include *Stuckenia pectinata L.* (Demirizen and Aksoy 2004; Marchand et al. 2014; Pell et al. 2013) and *Galium aparine L.* (Massa et al. 2010), and *Urtica dioica L.* (Balabanova et al. 2015). This study investigated the bioaccumulation of metal(loid)s, including arsenic and its species in plants (*Stuckenia pectinata L.*, *Galium aparine L.*, *Urtica dioica L.*) growing above banks of polluted the Bytomka River.

Plant materials were tested for metal and metalloid concentration, including the organic and inorganic arsenic species As(III), As(V), AsB, MMA, and DMA. PLI (Tomlinson et al. 1980) and *I*_geo_ (Müller 1979) indexes were applied in order to quantify the degree and origin of bottom sediment contamination by heavy metals. Methods of chemometric analysis were used to investigate the relationship between metals and metalloids contained in water, bottom sediment, and plants. In the analysis of experimental data, the concepts of (Dis)similarity Analysis using a matrix of distances (Zerzucha and Walczak 2012; Zerzucha et al. 2012), Cluster Analysis (CA) (Massart and Kaufman 1983; Smoliński et al. 2002; Dabioch et al. 2012) and Principal Component Analysis (PCA) (Joliffe 1986; Wold et al. 1987; Dabioch et al. 2013) were used.

## Materials and Methods

### Research Object and Sampling Area

The Bytomka River basin is located in the Silesian Voivodeship (southern Poland) and has its river head in the town of Bytom. It is currently not possible to locate its natural sources. The Bytomka River length is more than 20 km; its catchment area covers approximately 150 km^2^. The river is a right-bank tributary of the Kłodnica River. Currently, the river has no natural sources but is assumed to originate at the Karbowski Ditch (Rów Karbowski) through which the municipal and industrial wastewater is operated. The river flows through Ruda Śląska and Zabrze areas and reaches the city of Gliwice where it flows into the Kłodnica River (Sośnica district of Gliwice). Along its course, the Bytomka River flows through a strongly urbanized area and in each of the cities live from 150,000 to 200,000 inhabitants (https://en.wikipedia.org/wiki/Silesia). The population density in the Śląskie Voivodeship in 2010 was 376 people/km^2^ and was the highest among all Polish voivodships (https://www.slaskie.pl/content/struktura-demograficzna). The River flows through city centers, close to residential buildings, parks, and recreation areas.

The Bytomka River has a very poor river network and small natural catchment area. It is fed mainly with mine water, wastewater discharges from industrial plants, municipal wastewater, and rainwater. Through many ditches and collectors, significant amounts of heavily polluted wastewater are discharged into it, and this often constitutes 80–90% of its average flow. In Gliwice, the Bytomka River flows into the Kłodnica River, which flows into the Oder River.

**Fig. 1:**
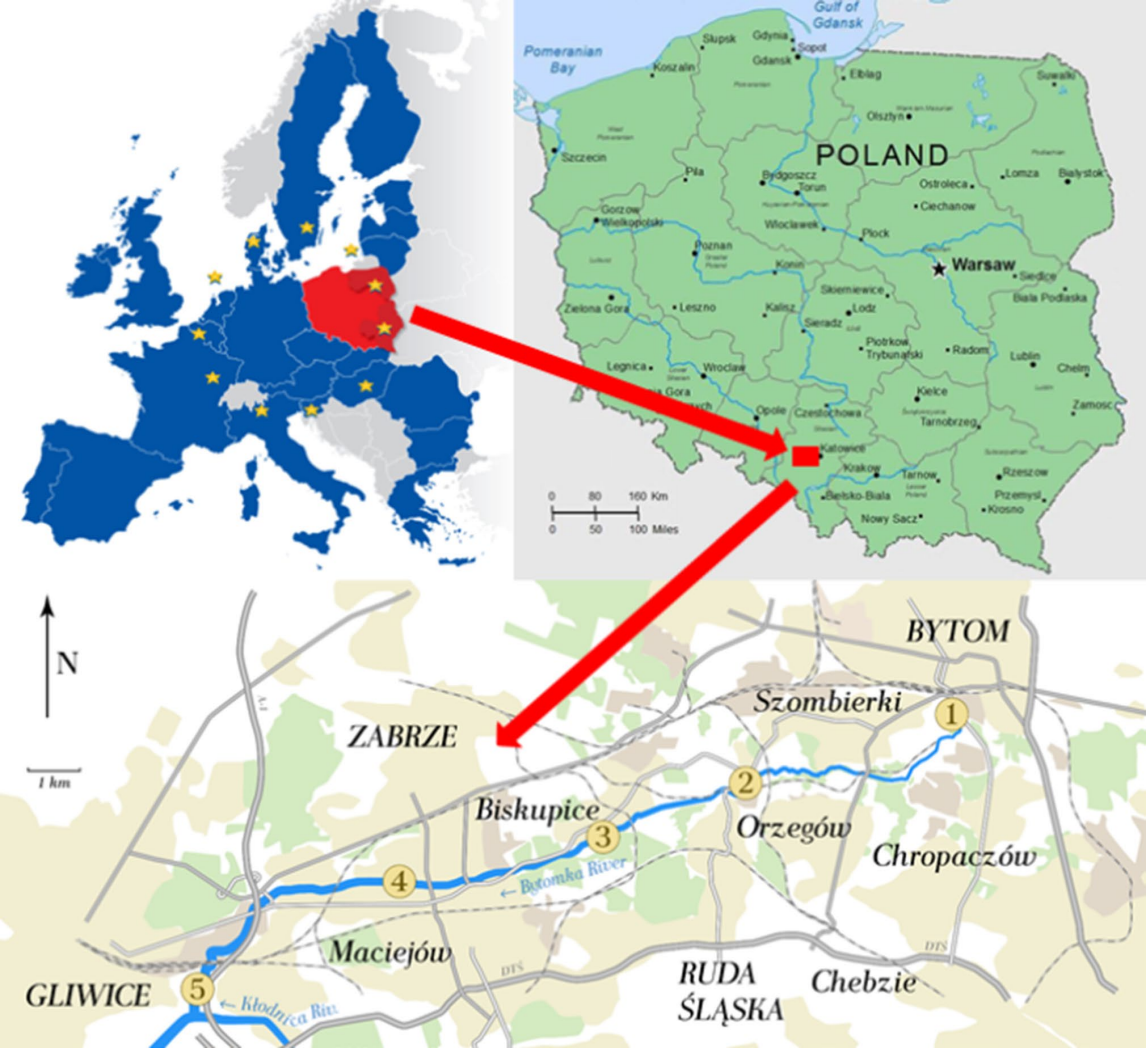
Plants, water, and bottom sediment sampling points on the Bytomka River; sampling points: 1—Bytom City (Szombierki), 2—Ruda Śląska City, Potokowa Street, 3—Zabrze City (Biskupice, Ruda Mine Dump), 4—Zabrze City, Nad Kanałem Street, 5—Gliwice City, Odrowążow Street

**Fig. 2:**
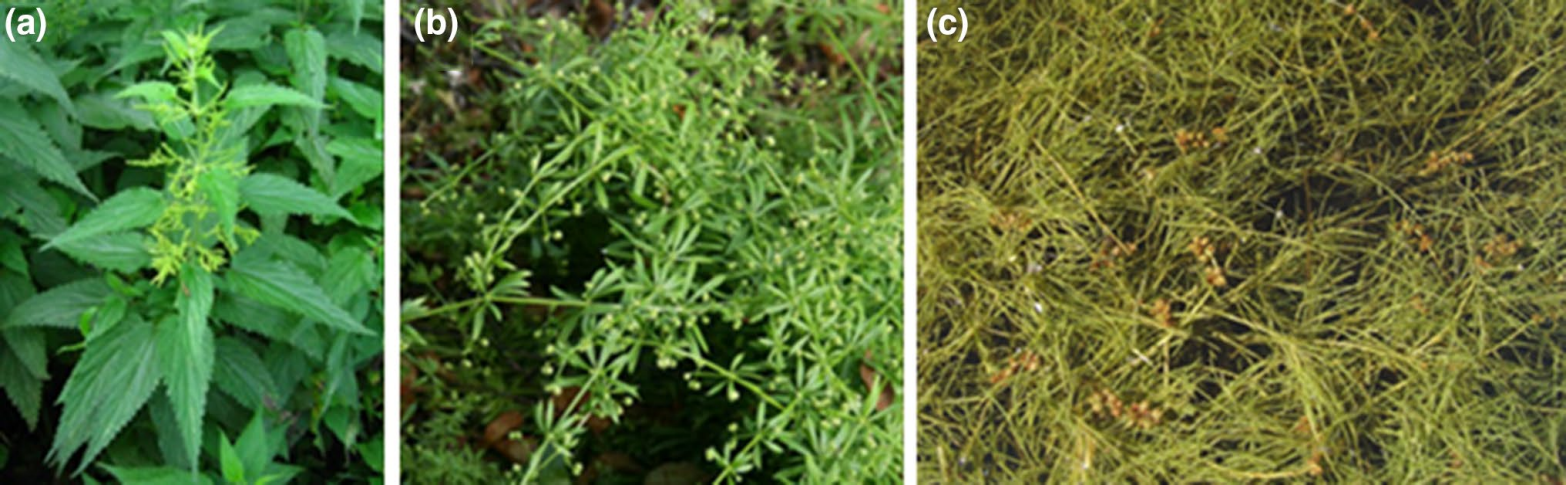
Photographs of studied plants: **a***Urtica dioica L.*, **b***Galium aparine L.*, **c***Stuckenia pectinata L*

Plant samples of *Urtica dioica L.*, *Galium aparine L.,Stuckenia pectinata L.* were collected directly at the riverbank in July 2014 from five sampling points shown in Fig. 1. Coastal plants were selected based on species availability at sampling sites B1-B5. Nevertheless, *Galium aparine L.* was not present at the second sampling point and *Stuckenia pectinata L.* was not present at the first and second points. Figure 2 shows photographs of studied plants. Water and bottom sediment samples were collected from April 2014 to March 2015 at the same sampling points of the Bytomka River.

### Sample Preparation and Research Methodology

Bottom sediments and surface waters were collected, prepared, and analysed similar to the earlier work (Jabłońska-Czapla et al. 2016). Water samples were taken from the middle part of the river current, dipping sampler below the water surface. Both water and bottom sediments were collected always in the same sampling points. Sediments were collected from the layer thickness of 0–5 cm. After being transported to the laboratory, the collected plant samples were washed with deionized water, cut, dried in air, and ground in a porcelain mortar in order to determine the total elements concentration. Then, the plant samples were combined with 5 ml of spectral HNO_3_ + 3 ml of water and mineralized in a MarsX microwave oven (digestion power—600 W, time—5 min up to 100 °C, next power—600 W, time—15 min up to 150 °C, stop time—10 min) and then the metal and metalloid content was determined using an ICP-MS spectrometer.

The plant samples for speciation analyses were washed with deionized water, cut, and stored at − 22 °C in a freezer for no longer than a month. Based on an in-depth literature analysis, the extraction of plant samples was not optimized. However, a method offering the highest arsenic extraction yield from the samples of the plant origin was selected (Zheng et al. 2003). A 1-g sample of plant material was extracted with 10 ml of a water:methanol solution in a ratio 1:9. HPLC-purity methanol was used. Each plant sample tested in triplicate and extracted with 2-hour shaking in a shaker (165 rpm). The extracted plant samples underwent filtration through a 0.22-μm syringe filter and were injected into the HPLC-ICP-MS system for the arsenic speciation analysis.

### Apparatus and Reagents

The in situ physicochemical data (temperature, pH, electrolytic conductivity (EC), and redox potential (Eh)) were collected using the CX-401 multiparameter meter (Elmetron, Poland) equipped with an ERH 111 glass electrode (Hydromet, Poland), an ERPt-111 platinum electrode (Hydromet, Poland), and a CD-2 conductometric sensor (Hydromet, Poland) with a built-in thermometer. EC and pH values were double-checked and the pH meter was calibrated with three pH buffer solutions (pH = 3.0, 7.0, and 9.0).

The Elan 6100 DRC-e ICP-MS spectrometer (PerkinElmer) was used for quantitative analyses of total metal/metalloid content in water, digested sediment, and plant samples, as well as plant and sediment extracts. The apparatus was equipped with a standard ICP quartz torch, cross-flow nebulizer, and nickel cones. Samples and standards were delivered with a peristaltic pump. The spectrometer was optimized daily with a 10-µg/L solution (Mg, Cu, Rh, Cd, In, Ba, Ce, Pb, and U) in 1% HNO_3_ Elan 6100 Setup/Stab./Masscal solution (PerkinElmer). The concentrations of ^51^V, ^53^Cr,^55^Mn, ^59^Co, ^60^Ni, ^65^Cu, ^66^Zn, ^69^Ga, ^75^As, ^85^Rb, ^88^Sr, ^98^Mo, ^107^Ag, ^114^Cd, ^130^Te, ^123^Sb ^138^Ba, ^205^Tl, ^208^Pb, and ^238^U were measured with the internal ^103^Rh standard. The arsenic (As(III), As(V), AsB, MMA, and DMA) species were determined with the HPLC-ICP-MS system. To separate the analytes, a speciation apparatus set was applied. It consisted of the HPLC chromatograph (PerkinElmer) equipped with Series 200 LC Peltier oven, Series 200 LC autosampler, and Series 200 LC gradient pump. The operating parameters of the ICP-MS spectrometer are given in the study (Jabłońska-Czapla 2015). The HPLC-ICP-MS separation was performed after optimization and applied a Hamilton PRP-X100 (100 mm × 4 mm, 10 µm) column at a temperature of 30 °C, with solutions A—20 mM NH_4_NO_3_ (pH = 8.7) and B—60 mM NH_4_NO_3_ (pH = 8.7) as the mobile phase, and the elution program (0–2.0 min 100% A; 2.0–3.0 min from 100% A to 100% B; 3.0–6.5 min 100% B; rinsing 6.5–9.5 min 100% A). The flow rate during the analysis and rinsing was 1.1 mL/min, and the volume of sample was 100 µL. The retention times were as follows: AB 1.84 min; As(III) 2.16 min; DMA 2.95 min; MMA 5.36 min; As(V) 6.17 min (Jabłońska-Czapla and Szopa 2016).

The following substances were used for analyses: ultra-pure ammonium nitrate (Merck, Darmstadt, Germany); sodium dihydrogen arsenate heptahydrate (Sigma-Aldrich, St. Louis, MO); sodium arsenite (Sigma-Aldrich); disodium methyl arsenate (Supelco, Bellefonte, PA); arsenobetaine (Sigma-Aldrich); and dimethylarsinic acid (Supelco). The calibration solutions were prepared each time through diluting suitable standard solutions on an analytical balance. The multielement standards no. XXI and VI (Merck) were used for determining total metal(loid)s content with the ICP-MS spectrometer. Solutions made from salts were used for calibration during quantitative determinations of the As species. All solutions and standards were prepared with the Milli-Q-Gradient ultra-pure deionized water (Millipore, Merck), whose electrolytic conductivity was < 0.05 μS/cm.

### Quality Control

The method validation was performed on the basis of the certified reference material (NIST 1643e Trace Elements in Water). The element’s concentrations were determined using an ICP-MS spectrometer with a low detection limit and amounted to the following: V—0.09; Mn—0.03; Co—0.002; Ni—0.024; Cu—0.064; Zn—0.181; As—0.096; Rb—0.003; Sr –0.008; Ag—0.002; Cd—0.040; Ba—0.010; Tl—0.002; Pb—0.036; Cr—0.013; and Sb—0.009 µg/L.

In order to validate the methodology, the total arsenic content analysis in the CRM NIST Tomato Leaves CRM 1573a sample was made. During the determination of arsenic species the following validation parameters were obtained: limit of detection (LOD) As(III)—0.08, As(V)—0.12, AB—0.16, MMA—0.08, DMA—0.09 [µg/L]; recovery As(III)—96, As(V)—104, AB—93, MMA—95, DMA—95%; uncertainty As(III)—12, As(V)—11, AB—17, MMA—12, DMA—11 [%]; RSD (relative standard deviation) repeatability As(III)—2.9, As(V)—2.4, AB—3.7, MMA—3.1, DMA—2.7%.

The recovery and the extraction efficiency (water/methanol extraction efficiency 9:1) also was checked using the certified reference material NIST Tomato Leaves 1573a, and it was 101.7% and 41.7%, respectively.

### Sediment Pollution Indices

The pollution load index (PLI) and geo-accumulation index (*I*_geo_) were employed to assess the pollution of metals in the sediment of the Bytomka River. The pollution load index was evaluated using the procedure of Tomlinson et al. (1980):

1 $${\text{PLI}} = \left( {{\text{CF}}1 \times {\text{CF}}2 \times {\text{CF}}3 \times \cdots \times {\text{CF}}n} \right)^{{\frac{1}{n}}}$$

where *n* denotes the number of metals and CF is the contamination factor, which is calculated according to the Eq. (2):

2 $${\text{CF}} = \frac{{C_{\text{Me}} }}{{C_{\text{Ref}} }}$$

where, *C*_Me_ is the metal concentration in polluted sediment and *C*_Ref_ is the metal reference concentration. The values of reference metal concentrations for bottom sediments of Polish rivers were taken from (Bojakowska and Sokołowska 1998).

PLI is an effective tool for preliminary assessment of the degree of contamination of bottom sediments. According to Chakravarty and Patgiri (2009), a PLI value > 1 means contamination of bottom sediment, whereas a PLI value < 1 means that bottom sediment is not contaminated by heavy metals.

The geo-accumulation index allows one to assess the level of bottom sediment pollution by individual heavy metals. Values of *I*_geo_ were calculated for ten metals (Ag, As, Ba, Cd, Co, Cr, Cu, Ni, Pb, and Zn) using the equation of Müller (1979):

3 $$I_{\text{geo}} = \log_{2} \frac{{C_{n} }}{{1.5B_{n} }}$$

where C_n_, is the measured concentration of heavy metals in bottom sediment, and B_n_, is the geochemical background value (Bojakowska and Sokołowska 1998). The multiplication factor 1.5 reduces possible variation of lithogenic effects. The *I*_geo_ is typically divided into six grades: *I*_geo_ = 0, background concentration; *I*_geo_ = (0–1), unpolluted; *I*_geo_ = (1–2), moderately polluted to unpolluted; *I*_geo_ = (2–3), moderately polluted; *I*_geo_ = (3–4), moderately to highly polluted; *I*_geo_ = (4–5), highly polluted; and *I*_geo_ > 5, very highly polluted (Wedepohl 1995).

### Chemometric Analysis

Measurement data concerning contents of selected chemical substances in water, bottom sediment, and plants has been pre-processed by centering and standardization:

4 $$x_{ij,a} = \frac{{\left( {x_{ij} - \overline{x}_{j} } \right)}}{{s_{j} }}$$

where *x*_*j*_ is the arithmetic mean of the *j*th column, *s*_*j*_ is the standard deviation of the *j*-th parameter, and *x*_*ij*_ and *x*_*ij,a*_ are the *i*-th parameter values before and after autoscaling, respectively. The data prepared in this way were subjected to the analysis of similarity using the similarity matrix and dendrograms (Dabioch et al. 2012; Jabłońska-Czapla et al. 2015). Chemometric analysis of environmental data also used the Principal Component Analysis, which allows one to examine the correlation relationships between the contents of elements in the natural environment (Dabioch et al. 2012, 2013).

#### (Dis)similarity Analysis

After pre-processing, the data underwent visualization by calculating the dissimilarity matrix **D** with the Euclidean distance as the measure of dissimilarity (Zerzucha et al. 2012). The calculations were made according to the formula proposed by Al-Kashi, whose matrix form was (Zerzucha and Walczak 2012):

5 $$\varvec{D}^{2} = \left( {diag\left( {\varvec{XX}^{T} } \right) \cdot 1^{T} + 1 \cdot diag\left( {\varvec{XX}^{T} } \right)^{T} } \right) - 2 \cdot \left( {\varvec{XX}^{T} } \right)$$

where **X** is a data matrix, **1**^T^ is a row vector with elements equal to one and diag is an operator extracting only the diagonal elements from the matrix represented as a column vector. To obtain matrix **D**, the root of the matrix **D**^2^ value, calculated according to the formula (5), had to be calculated.

#### Cluster Analysis

Hierarchical clustering analysis (Abonyi and Feil 2007; Kaufman and Rousseeuw 1990; Massart and Kaufman 1983; Vogt et al. 1987) may be applied to multidimensional data sets to study (dis)similarities of objects in the variable space or similarities of variables in the object space. Any agglomerative hierarchical clustering method is characterized by the similarity measure used and by the way the resulting subclusters are merged (linked). This method usually produces a dendrogram, or other types of tree diagrams, as the final output. On the *x* axis of the dendrogram, the indices of clustered objects (or variables) are displayed, whereas the *y* axis represents the corresponding linkage distances (or an adequate measure of similarity) between the two objects or clusters which are merged. The results of the cluster analysis are based on the Euclidean distance as a measure of similarity between the individual samples (variables) and the “Ward” linkage algorithm.

#### Principal Component Analysis

The Principal Component Analysis (PCA) is a method that allows one to examine the relationship between the studied parameters (content of chemical elements in the environmental materials under study) and between samples collected at selected points of the ecosystem (Wold et al. 1987; Joliffe 1986; Abdi and Williams 2010). In the PCA method, a matrix ***Y*** of the principal components is searched, which represents in the new space the input data matrix ***X***. To be able to determine the matrix ***Y***, it becomes necessary to calculate the transformation matrix ***W***. However, in the first step, a covariance matrix ***K*** that reflects linear relationships between variables should be calculated as follows:

6 $$\varvec{K} = \frac{1}{{\left( {n - 1} \right)}}\mathop \sum \limits_{i = 1}^{n} \left( {\varvec{x}_{i} - \overline{\varvec{x}} } \right)\left( {\varvec{x}_{i} - \overline{\varvec{x}} } \right)^{T} ;\quad \overline{\varvec{x}} = \frac{1}{n}\mathop \sum \limits_{i = 1}^{n} \varvec{x}_{i}$$

where ***x***_*i*_ are the data vectors that make up the matrix ***X***, and *n* is the number of vectors ***x***_*i*_ in the matrix ***X***. In the next step, the eigenvectors ***w***_*i*_ and eigenvalues *λ*_*i*_ of the covariance matrix are determined:

7 $$\varvec{Kw}_{i} = \lambda_{i} \varvec{w}_{i} .$$

Then, after sorting the eigenvalues from the largest to the smallest, we assign them the corresponding eigenvectors that are written in the form of the matrix ***W***. The first columns of this matrix are vectors defining directions in space that differentiate data the most. The eigenvectors matrix ***W*** is a sought-after transformation matrix that is used to determine the matrix of the principal components ***Y***, representing data in the new space:

8 $$\varvec{Y} = \varvec{XW};\quad \varvec{W} = \left[ {\varvec{w}_{1} , \varvec{w}_{2} , \ldots , \varvec{w}_{n} } \right]$$

The eigenvalues of the covariance matrix are the variances of the principal components that indicate which major components contain the largest percentage of information on the input data set.

## Results and Discussion

### Total Metal(loid)s Concentration

**Fig. 3:**
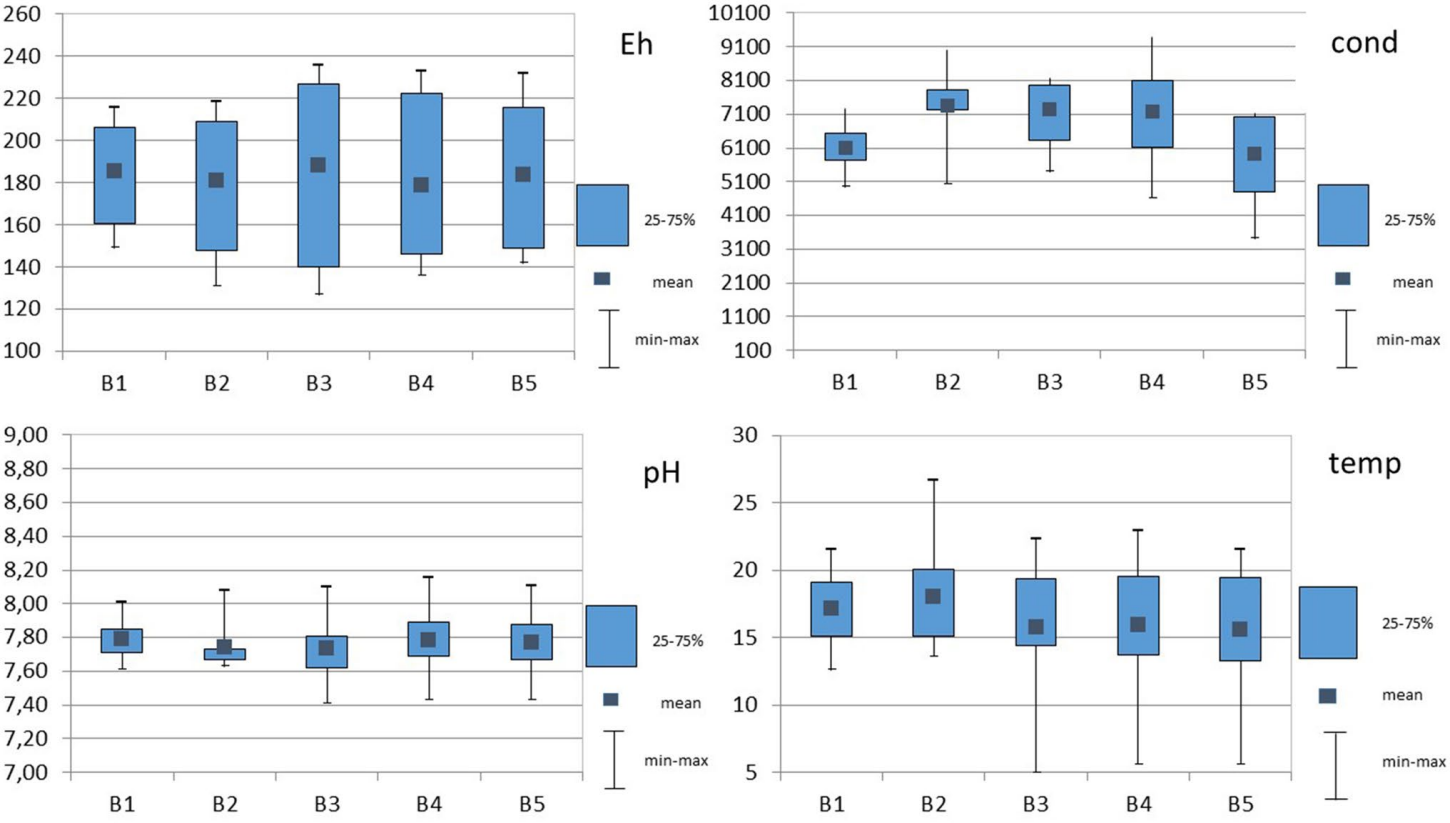
Box plots of pH, conductivity [µS] (cond), temperature [°C] (temp), and redox potential (Eh) data for Bytomka River monitoring (5 sampling points B1-B5, samples taken from April 2014 to March 2015 Bytomka River water)

The physicochemical conditions prevailing in the Bytomka River are shown in Fig. 3. Tables 1 and 2 show the total content of metal/metalloids in plant, water, and bottom sediment samples of the Bytomka River. Already, preliminary research results showed that plants growing on the banks of the Bytomka River strongly accumulated heavy metals.

**Table 1:** Total metal/metalloids concentration in Nettle (*Urtica dioica L.*), Goosegrass (*Galium aparine L.*), Sago pondweed (*Stuckenia pectinata L.*)

Element (mg/kg)	LOD	U (%)	Sago pondweed *(Stuckenia pectinata L.)*					Goosegrass *(Galium aparine L.)*					Nettle (*Urtica dioica L*.)				
			B1	B2	B3	B4	B5	B1	B2	B3	B4	B5	B1	B2	B3	B4	B5
V	0.09	10	N/A	N/A	5.55 ± 0.61	0.45 ± 0.05	1.48 ± 0.16	0.30 ± 0.03	N/A	0.69 ± 0.08	0.44 ± 0.05	2.05 ± 0.23	0.79 ± 0.09	2.80 ± 0.31	0.36 ± 0.04	1.61 ± 0.18	0.62 ± 0.07
Mn	0.03	10	N/A	N/A	1719 ± 172	3.15 ± 0.32	43.9 ± 4.39	15.9 ± 1.6	N/A	24.9 ± 2.5	38.8 ± 3.9	2.67 ± 0.27	39.5 ± 3.9	283 ± 28	39.1 ± 3.9	572 ± 57	34.9 ± 3.49
Co	0.07	10	N/A	N/A	6.71 ± 0.67	0.21 ± 0.02	0.31 ± 0.03	0.15 ± 0.02	N/A	0.24 ± 0.02	0.16 ± 0.02	4.54 ± 0.45	0.33 ± 0.03	1.44 ± 0.14	0.15 ± 0.02	2.75 ± 0.28	0.24 ± 0.02
Ni	0.11	21	N/A	N/A	20.9 ± 4.39	2.63 ± 0.55	3.17 ± 0.67	1.70 ± 0.36	N/A	3.71 ± 0.78	0.64 ± 0.13	10.1 ± 2.1	2.23 ± 0.47	17.3 ± 3.6	1.23 ± 0.26	7.78 ± 1.63	2.42 ± 0.51
Cu	0.11	10	N/A	N/A	11.0 ± 1.1	6.03 ± 0.60	9.21 ± 0.92	8.61 ± 0.92	N/A	9.07 ± 0.91	3.86 ± 0.39	8.18 ± 0.82	9.51 ± 0.95	11.5 ± 1.2	9.53 ± 0.95	7.97 ± 0.80	6.12 ± 0.61
Zn	0.18	31	N/A	N/A	438 ± 136	73 ± 22	80 ± 25	88.3 ± 27.4	N/A	158 ± 49	26.1 ± 8.1	255 ± 79	49.6 ± 15.4	193 ± 60	228 ± 70	399 ± 124	101 ± 31.3
As	0.09	29	N/A	N/A	65.4 ± 19	3.85 ± 1.12	10.6 ± 3.07	2.23 ± 0.65	N/A	10.5 ± 3.0	4.25 ± 1.23	1.73 ± 0.50	3.48 ± 1.01	16.8 ± 4.9	6.54 ± 1.90	29.9 ± 8.67	4.09 ± 1.19
Rb	0.003	14	N/A	N/A	20.7 ± 2.9	9.70 ± 1.36	12.8 ± 1.79	15.5 ± 2.2	N/A	16.7 ± 2.3	11.4 ± 1.6	16.4 ± 2.3	15.0 ± 2.1	19.3 ± 2.7	17.3 ± 2.4	23.5 ± 3.29	24.4 ± 3.42
Sr	0.01	11	N/A	N/A	165 ± 18.1	0.24 ± 0.03	101 ± 11	93.1 ± 10.2	N/A	190 ± 21	146 ± 16	0.20 ± 0.02	102 ± 11	103 ± 11	121 ± 13	111 ± 12.2	79.3 ± 8.7
Ag	0.002	10	N/A	N/A	0.19 ± 0.02	0.06 ± 0.01	0.17 ± 0.02	0.08 ± 0.01	N/A	0.50 ± 0.05	0.17 ± 0.02	0.02 ± 0.002	0.12 ± 0.01	0.39 ± 0.04	0.25 ± 0.03	0.06 ± 0.01	0.11 ± 0.01
Cd	0.01	10	N/A	N/A	1.01 ± 0.01	0.33 ± 0.03	0.40 ± 0.04	0.54 ± 0.05	N/A	0.28 ± 0.03	0.14 ± 0.01	0.20 ± 0.02	0.24 ± 0.02	1.93 ± 0.19	1.88 ± 0.19	0.24 ± 0.02	0.53 ± 0.05
Ba	0.1	15	N/A	N/A	62.4 ± 9.4	0.77 ± 0.12	19.6 ± 2.94	223 ± 33	N/A	32.3 ± 4.8	28.2 ± 4.2	22.3 ± 3.34	27.6 ± 4.1	29.5 ± 4.4	80.4 ± 12.1	29.4 ± 4.4	79.2 ± 11.9
Tl	0.004	28	N/A	N/A	0.26 ± 0.07	0.03 ± 0.01	0.30 ± 0.08	0.32 ± 0.09	N/A	0.78 ± 0.22	0.13 ± 0.04	0.04 ± 0.01	0.39 ± 0.11	0.44 ± 0.12	0.92 ± 0.26	0.21 ± 0.06	0.50 ± 0.14
Pb	0.09	12	N/A	N/A	73.2 ± 8.8	3.11 ± 0.37	7.64 ± 0.92	7.51 ± 0.90	N/A	6.57 ± 0.79	2.48 ± 0.30	35.1 ± 4.2	8.40 ± 1.01	56.7 ± 6.8	4.44 ± 0.53	34.4 ± 4.13	4.59 ± 0.55
Cr	0.11	23	N/A	N/A	62.2 ± 14.3	7.84 ± 1.8	15.5 ± 3.6	4.51 ± 1.03	N/A	12.5 ± 2.9	5.85 ± 1.34	21.8 ± 5.0	10.2 ± 2.35	76.7 ± 17.6	4.50 ± 1.03	8.47 ± 1.95	9.09 ± 2.09
Sb	0.01	28	N/A	N/A	0.71 ± 0.19	0.10 ± 0.03	0.15 ± 0.04	0.13 ± 0.04	N/A	0.10 ± 0.03	0.04 ± 0.01	0.50 ± 0.14	0.24 ± 0.07	0.45 ± 0.13	0.03 ± 0.01	0.42 ± 0.12	0.04 ± 0.01

B1–B5—sampling points; *N/A* not present at sampling point (July 2014); *LOD* limit of detection; *U* uncertainty

**Table 2:** Total metal/metalloids concentration in water and bottom sediment samples (July 2014)

Element	LOD	U (%)	B1		B2		B3		B4		B5	
			Water (µg/L)	Sediment (mg/kg)	Water (µg/L)	Sediment (mg/kg)	Water (µg/L)	Sediment (mg/kg)	Water (µg/L)	Sediment (mg/kg)	Water (µg/L)	Sediment (mg/kg)
V	0.09	10	8.44 ± 0.93	74.1 ± 8.2	7.64 ± 0.84	26.2 ± 2.9	7.29 ± 0.81	131 ± 14	6.51 ± 0.72	46.5 ± 5.1	5.65 ± 0.62	24.8 ± 2.7
Mn	0.03	10	346 ± 35	1721 ± 172	326 ± 32	1256 ± 126	352 ± 35	1644 ± 164	324 ± 32	610 ± 61	250 ± 25	658 ± 66
Co	0.07	10	1.82 ± 0.18	28.9 ± 2.9	1.23 ± 0.12	7.65 ± 0.77	1.15 ± 0.12	22.9 ± 2.3	1.12 ± 0.11	9.73 ± 0.97	0.98 ± 0.10	3.35 ± 0.34
Ni	0.11	21	7.53 ± 1.58	71.4 ± 15.0	8.94 ± 1.88	57.4 ± 12.0	10.3 ± 2.2	69.4 ± 14.6	9.96 ± 2.09	26.4 ± 5.5	8.24 ± 1.73	9.37 ± 1.97
Cu	0.11	10	3.26 ± 0.33	172 ± 17.2	3.67 ± 0.37	39.1 ± 3.9	2.83 ± 0.28	172 ± 17	2.54 ± 0.53	69.9 ± 14.7	3.59 ± 0.75	27.1 ± 5.7
Zn	0.18	31	36.0 ± 11.2	2519 ± 781	57.2 ± 17.7	5333 ± 1653	67.1 ± 20.8	10101 ± 3131	63.4 ± 19.6	1274 ± 395	65.4 ± 20.3	551 ± 171
As	0.09	29	7.64 ± 2.22	54.7 ± 15.9	10.2 ± 3.0	12.6 ± 3.6	10.0 ± 2.9	67.4 ± 19.5	9.23 ± 2.68	16.2 ± 4.7	6.67 ± 1.93	5.08 ± 1.47
Rb	0.003	14	39.8 ± 5.6	65.2 ± 9.1	44.8 ± 6.3	52.3 ± 7.3	40.62 ± 5.69	55.9 ± 7.83	36.4 ± 5.1	98.9 ± 13.9	30.0 ± 4.2	33.9 ± 4.8
Sr	0.01	11	1989 ± 219	355 ± 39	2850 ± 313	179 ± 20	2369 ± 261	306 ± 34	2056 ± 226	159 ± 18	1464 ± 161	78.7 ± 8.7
Ag	0.002	10	0.22 ± 0.02	4.08 ± 0.41	0.09 ± 0.01	4.08 ± 0.41	0.01 ± 0.001	9.97 ± 1.00	0.06 ± 0.01	2.89 ± 0.29	0.04 ± 0.01	0.62 ± 0.06
Cd	0.01	10	0.29 ± 0.03	11.4 ± 1.1	0.13 ± 0.01	4.18 ± 0.42	0.35 ± 0.04	40.2 ± 4.0	0.12 ± 0.01	4.57 ± 0.46	0.34 ± 0.03	3.08 ± 0.31
Ba	0.1	15	80.9 ± 12.1	412 ± 62	50.4 ± 7.6	484 ± 73	53.9 ± 8.1	1478 ± 222	50.4 ± 7.6	631 ± 95	49.4 ± 7.4	789 ± 118
Tl	0.004	28	0.07 ± 0.02	1.60 ± 0.45	0.07 ± 0.02	1.33 ± 0.37	0.06 ± 0.02	6.36 ± 1.78	0.06 ± 0.02	1.29 ± 0.36	0.05 ± 0.01	0.86 ± 0.24
Pb	0.09	12	5.30 ± 0.64	1541 ± 185	1.27 ± 0.15	346 ± 42	7.54 ± 0.91	2395 ± 287	1.22 ± 0.15	477 ± 57	8.01 ± 0.96	62.2 ± 7.5
Cr	0.11	23	25.2 ± 5.8	99.7 ± 22.9	23.6 ± 5.43	32.7 ± 7.5	21.6 ± 5.0	185 ± 43	18.6 ± 4.3	76.2 ± 17.5	14.0 ± 3.2	51.9 ± 11.9
Sb	0.01	28	1.77 ± 0.50	4.49 ± 1.26	1.38 ± 0.39	1.61 ± 0.45	1.56 ± 0.44	8.39 ± 2.35	1.55 ± 0.43	2.13 ± 0.60	1.40 ± 0.39	0.79 ± 0.22

B1–B5 Bytomka River sampling points; *LOD* limit of detection; *U* uncertain

**Fig. 4:**
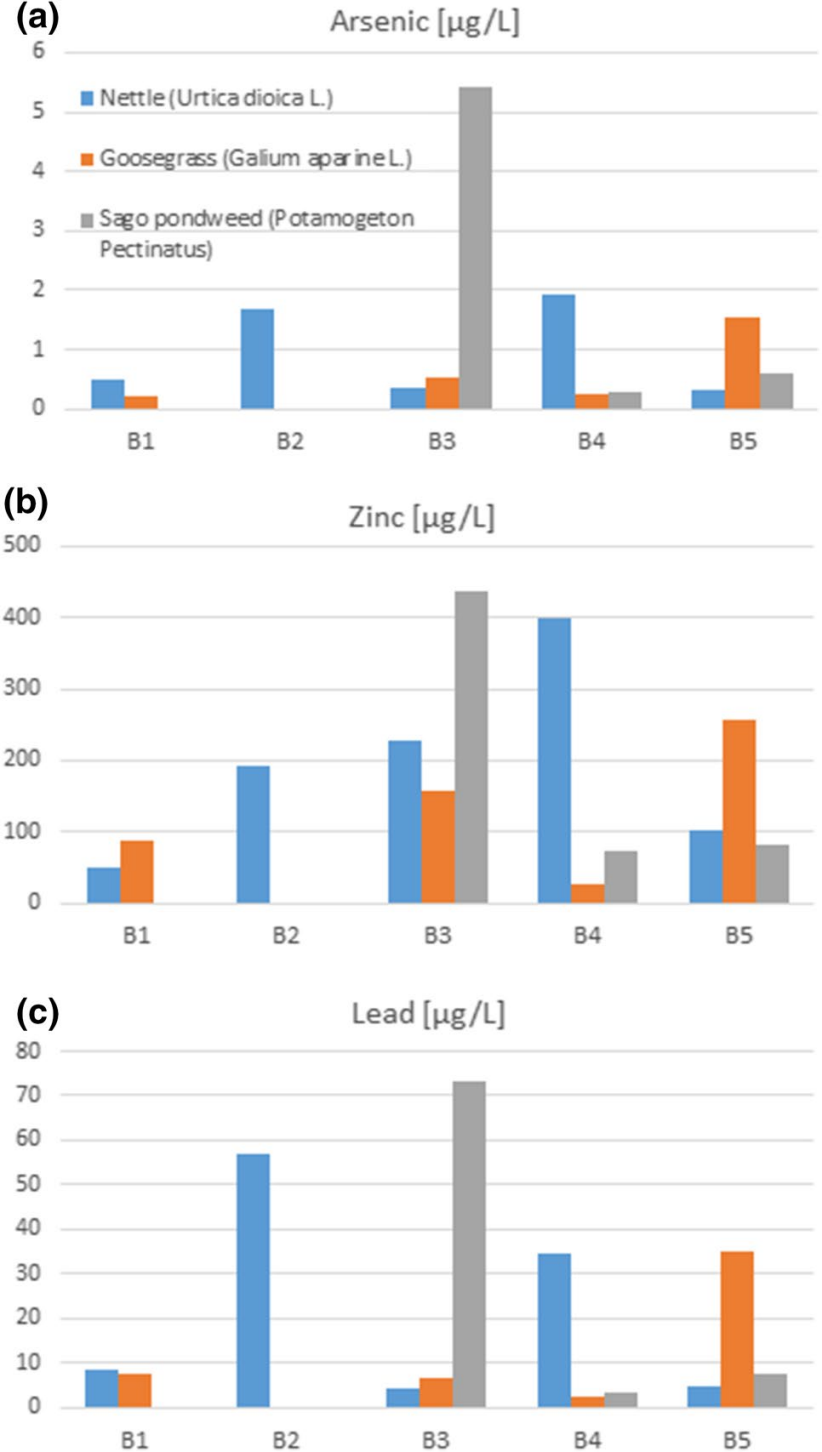
Arsenic (**a**), zinc (**b**), and lead (**c**) content in coastal plants growing on the Bytomka River; (B1–B5—sampling points)

**Fig. 5:**
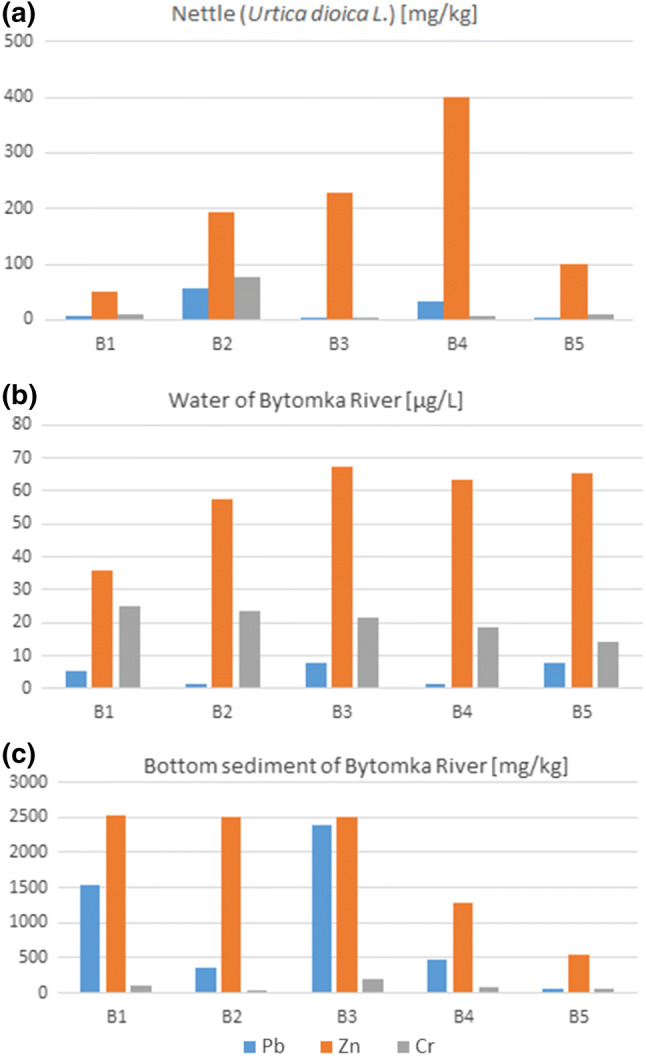
Comparison of lead, zinc, and chromium content in **a** Nettle (*Urtica dioica L.*), **b** water, **c** bottom sediment of the Bytomka River; (B1–B5—sampling points)

Among the studied plants, the highest concentrations of arsenic, lead, and zinc were found in Sago pondweed (*Stuckenia pectinata L.*) at the third sampling point B3 located near the post-mining dump “Hałda Ruda” in Zabrze (Fig. 4). This correlates with the significant Bytomka River bottom sediment’s zinc contamination at this sampling point, whose concentration was as high as 10.1 g/kg (Fig. 5). The concentration of zinc in Nettle (*Urtica dioica L.*) and Goosegrass (*Galium aparine L*.) increased with the flow of the river. The highest concentration of zinc in Nettle (*Urtica dioica L*.) was found at the fourth sampling point in Zabrze. The Bytomka River flowing through the Upper Silesian agglomeration is enriched with metals and metalloids carrying these pollutants to the Kłodnica River, which in turn pollutes the main Polish river: the Odra River. Earlier research results revealed that the waste stored at “Hałda Ruda” largely affected the increase in the heavy metal contents in the Bytomka River bottom sediments as their lowest and highest concentrations were observed in the samples collected above the dump (B1) and below it (B3), respectively (Jabłońska-Czapla et al. 2014).

As our research has shown, the Bytomka River is heavily polluted with cadmium, zinc, and lead (Table 2). Due to the permissible content of heavy metals in bottom sediments according to the LAWA classification (LAWA 1998; Parzych 2016), bottom sediments of the Bytomka River should be included in the IV class of purity.

The examined plants were characterized by a low antimony content, which correlates with a relatively low concentration of this element in the Bytomka River waters. Antimony like arsenic in plants is characterized by a similar degree of bioaccumulation and behaviour. It also is accumulated by plants; it can be toxic to them and inhibit their development, and the toxic antimony concentration in plants is 5–10 mg/kg (Seńczuk 1990). Nettle (*Urtica dioica L.*) is a plant that is most resistant to heavy metals present at each sampling point.

Coastal plants contained significant amounts of chromium, with the largest bioaccumulation demonstrated by Nettle (*Urtica dioica L*.) and Sago pondweed (*Stuckenia pectinata L.*); in the extreme case in the bank of the Bytomka River, it was 76.74 and 62.20 mg/kg Cr, respectively.

In terms of arsenic concentration from examined plants, Sago pondweed (*Stuckenia pectinata L.*) showed the highest bioaccumulation (5.42 mg/kg). This content indicates the degree of the Bytomka River pollution. This was confirmed by previous studies of Pell et al. (2013), who found that the arsenic content in Sago pondweed (*Stuckenia pectinata L.*) growing in Chile in the Loa River Basin was 20 mg/kg. Research performed by Baldantoni and others (2003) showed that Sago pondweed (*Stuckenia pectinata L.*) growing on Averno Lake in Italy was characterized by a maximum chromium concentration of 3 mg/kg in the leaves. Other studies conducted in Turkey showed that the concentration of chromium in Sago pondweed (*Stuckenia pectinata L.*) growing above Sultan Marsh (Kayseri, Turkey) was almost 8 mg/kg (Demirizen and Aksoy 2004). In northern Italy, Massa et al. (2010) conducted research on the content of metals and metalloids in plants, including Goosegrass (*Galium aparine L*.), which showed 1.7 mg/kg of Cr and 0.3 mg/kg of As in the leaf and 181.5 of mg/kg Cr and 13.9 mg/kg of As in the root.

The results obtained in the present study of metal and metalloid concentration in plants are an average, because the plant samples were examined in their entirety without separation into roots, stems, or leaves. However, it turns out that in terms of the arsenic and chromium content, Goosegrass (*Galium aparine L*.) growing by the banks of the Bytomka River was characterized by a lower degree of accumulation of these pollutants.

### Impact of the Physicochemical Parameters on the Concentration of Metal(loid)s in Bytomka River Waters

The physicochemical conditions of the Bytomka River are shown in Fig. 3. The influence of physicochemical parameters on the variability of an element form’s concentration is complex and depends on the pH, conductivity, redox potential, and temperature. A pH decrease led to the dissolution of carbonates and hydroxides and the metal ions were displaced with hydrogen ions. An increase in the salt content of water caused a shift of the sorption isotherm to the higher pH direction or led to the formation of soluble metal-chloride ion complexes. A change in the redox potential caused a change in the metal binding forms in the solid phase and a pH drop, which increased the metal mobility (Jabłońska-Czapla 2015). The pH and temperature values remained at a similar level throughout the river course. The highest redox potential value and the largest spread of the results was found at the third collection point, B3, located near the post-mining waste dump “Hałda Ruda”. The Bytomka River waters were characterized by high conductivity, which amounted to as much as 9380 µS. The highest conductivity value observed at the third and fourth sampling points B3 and B4 (in Zabrze) related to with the highest ion concentrations (mainly chlorides and sulphates). Such a finding confirms the high salt content in the supplying mine water. Mine water discharges in the Upper Silesian Rivers are a big problem not only in this area (Policht-Latawiec and Kapica 2013). In addition, the concentration of lead, cadmium, or lead in both water and bottom sediment was highest at the third B3 sampling point (Fig. 2). Interestingly, chromium concentration in the Bytomka River waters decreased slightly along the river course. In the case of arsenic, the highest concentration of this element was found at the third sampling point in bottom sediment. At the same time, Bytomka River water contained the most arsenic at the second and third point. The high amount of arsenic in bottom sediments and water has a strong impact on the content of this metalloid in plants. The highest concentrations of arsenic at the B3 sampling point were found in Sago pondweed (*Stuckenia pectinata L.*) and Goosegrass (*Galium aparine L*.) and were 65.43 and 10.54 mg/kg, respectively. Only the Nettle (*Urtica dioica L*.) accumulated more arsenic in other collection points, such as B3. At B4, the arsenic concentration in Nettle (*Urtica dioica L*.) was 29.89 mg/kg, whereas at B3, it was only 6.54 mg/kg. This is probably related to stronger soil contamination of Bytomka River banks on which the Nettle (*Urtica dioica L.*) grew.

### Arsenic Speciation

**Fig. 6:**
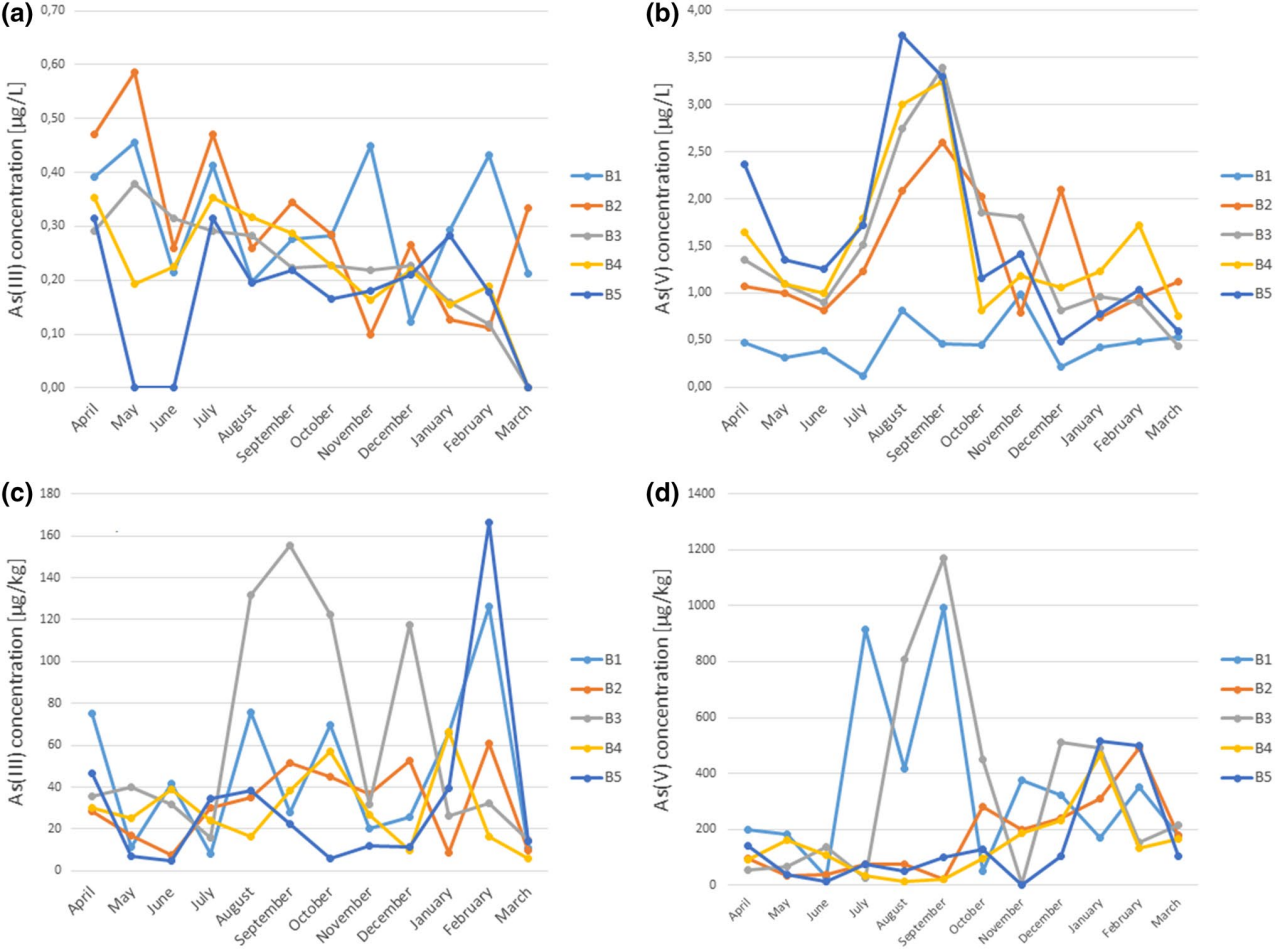
Arsenic species concentration in water: **a** As(III) [µg/L], **b** As(V) [µg/L] and bottom sediments: **c** As(III) [µg/kg], **d** As(V) [µg/kg] of the Bytomka River

After extraction with a 9:1 water–methanol solution, the plant samples were subjected to speciation analysis using the HPLC-ICP-MS system. Table 3 presents the content of arsenic species in the tested plants growing on the Bytomka River. Five arsenic species were determined, and it was shown that coastal plants contain the largest amounts of As(V) and As(III), as well as methyl derivatives, such as MMA and DMA. AsB was marked only in one plant sample (Table 3). Plants partially metabolize arsenic into its methyl derivatives, especially in strong pressure conditions. It is a kind of a defence mechanism against environmental pollution. In the methylation processes, plants transform inorganic ionic arsenic forms into MMA or DMA. The polluted environment in which the plants grow has an important impact on the content of metals and metalloids in coastal Bytomka plants. High arsenic content in the bottom sediment (B3—67.4 mg/kg) as well as in water (maximum 18 μg/L) of the river over which the plants grow significantly increases the concentration of this metalloid in plants. Plants often are able to change their tolerance to a given element in the presence of elevated concentrations in the environment. The plant populations growing on polluted soils are able to adapt their tolerance of the current metal content in the soil (Szakova et al. 2009, 2011). In the Bytomka River water and sediments, the oxidized As form was predominant; neither AsB nor DMA was found. As(V) dominated quantitatively in the Bytomka River sediments, and its mean concentration was 139.23 μg/kg. The organic species occurred extremely rarely. DMA was not found at all, while MMA was only observed in three bottom sediment samples. It is generally known that under oxidative conditions (i.e., when Eh is positive and pH < 6.9) As(V) is the dominant form. At higher pH, As(III) (reduced form) dominates. As(V) dominates in a highly acidic environment. When pH is less than 9.2 and under reducing conditions (negative Eh), As occurs as As(III) (Selene et al. 2003). As the redox conditions of river systems are generally oxic, the main As species in the river water and particulate matters (and sediments) are pentavalent (Jabłońska-Czapla and Zerzucha 2019). Figure 6 shows the seasonal dynamics of As(III) and As(V) concentration in water and bottom sediments. The increase in the As(V) concentration along the river course was characteristic for the Bytomka River. Flowing through the most industrial part of the Upper Silesian region, the river became increasingly polluted, with the highest As(V) concentration observed at the B5 sampling point (final sampling point) at the river mouth, where the Bytomka River joins the Kłodnica River. The seasonality of changes in the As(V) concentration also was observed, which consisted in the occurrence of a rising trend from spring to summer. The maximum concentration was obtained in August and September 2014, and a decrease in the ion concentration in the Bytomka River water samples was observed between September 2014 and March 2015. The highest As(V) concentration occurred in August 2014 at the B5 sampling point (3.73 μg/L). The As(III) concentration was maximum 0.59 μg/L, but its mean value was 0.26 μg/L (Fig. 6). Its highest concentration occurred at the B1 and B2 sampling points (Bytom and Ruda Śląska).

**Table 3:** Arsenic species in Nettle (*Urtica dioica L.*), Goosegrass (*Galium aparine L.*), and Sago pondweed (*Stuckenia pectinata L.*)

Arsenic species in plant samples (mg/kg)	LOD	U (%)	Sampling point				
			B1	B2	B3	B4	B5
*Nettle* (*Urtica dioica L.)*							
As(V)	0.12	11	3.36 ± 0.37	16.5 ± 1.8	5.62 ± 0.62	26.7 ± 2.9	3.09 ± 0.34
As(III)	0.08	12	0.16 ± 0.02	<LOD	0.25 ± 0.03	2.00 ± 0.24	0.43 ± 0.05
MMA	0.08	12	<LOD	<LOD	<LOD	<LOD	<LOD
DMA	0.09	11	<LOD	<LOD	0.24 ± 0.03	<LOD	<LOD
AsB	0.16	17	<LOD	<LOD	<LOD	<LOD	<LOD
*Goosegrass (Galium aparine L.)*							
As(V)	0.12	11	1.94 ± 0.21	N/A	5.22 ± 0.57	0.34 ± 0.04	1.21 ± 0.13
As(III)	0.08	12	0.44 ± 0.05	N/A	2.24 ± 0.27	1.85 ± 0.22	0.38 ± 0.05
MMA	0.08	12	<LOD	N/A	<LOD	2.06 ± 0.25	<LOD
DMA	0.09	11	0.41 ± 0.05	N/A	<LOD	<LOD	<LOD
AsB	0.16	17	<LOD	N/A	0.92 ± 0.16	<LOD	<LOD
*Sago pondweed (Stuckenia pectinata L.)*							
As(V)	0.12	11	N/A	N/A	63.7 ± 7.0	3.08 ± 0.34	8.56 ± 0.94
As(III)	0.08	12	N/A	N/A	2.00 ± 0.24	0.35 ± 0.04	0.49 ± 0.06
MMA	0.08	12	N/A	N/A	<LOD	0.28 ± 0.03	<LOD
DMA	0.09	11	N/A	N/A	0.82 ± 0.09	0.35 ± 0.04	<LOD
AsB	0.16	17	N/A	N/A	<LOD	<LOD	<LOD

B1–B5 sampling points (July 2014); *N/A* not found at the sampling point; *LOD* limit of detection; *U* uncertainty

### Pollution Load Index and *I*_geo_

The PLI and *I*_geo_ indices were determined for each of the bottom sediment sampling points of the Bytomka River. This allows one to estimate the degree of contamination of the examined ecosystem with heavy metals (Table 4).

**Table 4:** PLI and *I*_geo_ values calculated for each of the five bottom sediment sampling points

Sampling point	PLI	*I*_geo_
1	6.13	Ag = 1.44; As = 1.87; Ba = 1.46; Cd = 2.93; Co = 0.95
		Cr = 0.41; Cu = 1.52; Ni = 1.57; Pb = 5.10; Zn = 3.07
2	2.97	Ag = 1.44; As = − 0.31; Ba = 1.69; Cd = 1.48; Co = − 0.97
		Cr = − 1.20; Cu = − 0.62; Ni = 1.26; Pb = 2.94; Zn = 4.15
3	10.98	Ag = 2.73; As = 2.17; Ba = 3.30; Cd = 4.74; Co = 0.61
		Cr = 1.30; Cu = 1.52; Ni = 1.53; Pb = 5.73; Zn = 5.07
4	3.00	Ag = 0.95; As = 0.11; Ba = 2.07; Cd = 1.61; Co = − 0.62
		Cr = 0.02; Cu = 0.22; Ni = 0.14; Pb = 3.41; Zn = 2.09
5	1.20	Ag = − 1.27; As = − 1.56; Ba = 2.40; Cd = 1.04; Co = − 2.16
		Cr = − 0.53; Cu = − 1.15; Ni = − 1.36; Pb = 0.47; Zn = 0.88

The values obtained clearly indicate significant pollution of the examined ecosystem with heavy metals. The index PLI indicates that there is significant contamination with heavy metals at each of the examined ecosystem points of the Bytomki River. The highest level of contamination was recorded for the third sampling point (the PLI index reaches the maximum value of 10.98), where the *I*_geo_ index pointed to high contamination by lead, zinc, cadmium, barium, silver, and arsenic. Equally high lead impregnation was noted for the first sampling point. There was the smallest contamination with heavy metals for the fifth sampling point. The index *I*_geo_ pointed to an average contamination with barium compounds for this sampling point. The earlier study of the bioavailability and mobility of elements using RAC (Risk Assessment Code) showed that Zn and Pb occurred in the bottom sediments in the most mobile forms (Jabłońska-Czapla et al. 2014). They demonstrated the highest content in the first three fractions, which are bound to the sediments in the most unstable way. For this reason, they are the most dangerous for the environment. The fact that Zn and Pb carbonates form easily can be attributed to the characteristic physical and chemical conditions of the Bytomka River. The RAC values for these analytes varied between 30 and 55% and even for this reason the Bytomka River sediments can be classified as extremely dangerous for the environment. Among all studied elements, As was (to the largest degree) bound to the ion-exchange fraction, which is the most easily available for the ecosystem. Arsenic can pass from the solid phase into water when the water ionic content changes as a result of the sorption–desorption balance shift. Even though As demonstrated increased mobility, its RAC value did not exceed 35% (Jabłońska-Czapla et al. 2014).

### Chemometric Analysis

#### (Dis)similarity Analysis

**Fig. 7:**
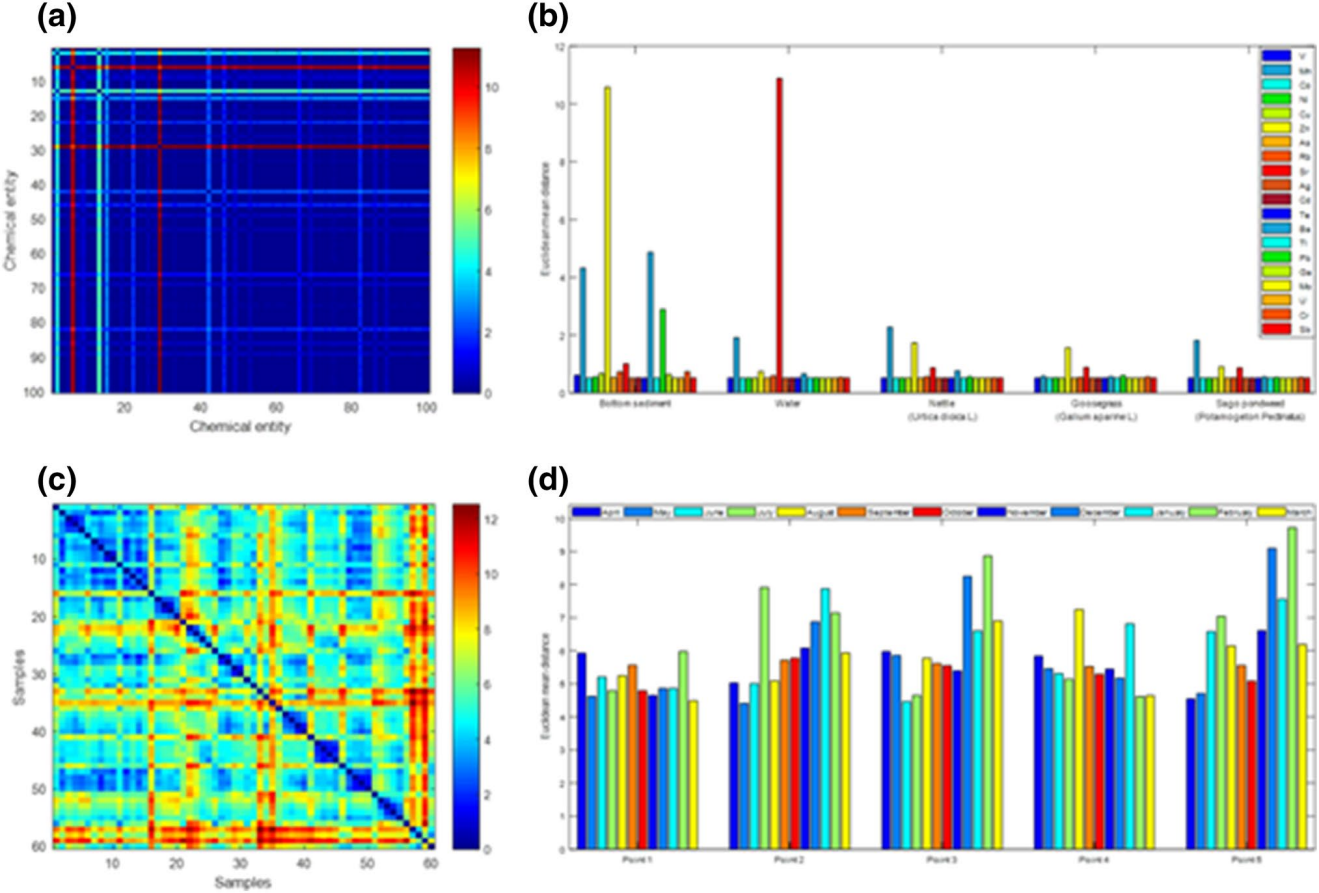
**a** Dissimilarity matrix **D** calculated with the Euclidean distance (the concentration of the studied chemical elements in bottom sediment, water, and plant material measured in the month of July). (**b**) Detection of chemical parameters exhibiting the greatest variability depending on the sampling point (the concentration of the studied chemical elements in bottom sediment, water, and plant material measured in the month of July). **c** Dissimilarity matrix **D** calculated with the Euclidean distance (the concentration of the studied chemical elements in water measured in 5 measurement points within 12 months). **d** Detection of the greatest variability of chemical parameters depending on the time and place of sampling (concentration of tested chemical elements in water measured at 5 measuring points within 12 months)

The determined dissimilarity matrix (Fig. 7a) helped to examine the data structure and determine which of the tested chemical parameters showed the greatest variability depending on the sampling point (Fig. 7b). In the case of water, the highest variability of the content has been observed for strontium, whose elevated levels of variability also were observed for bottom sediment as well as plant material. Another element showing significant variability in the water samples collected was manganese for which equally high variability was observed in the case of bottom sediment and in plant material with the exception of *Galium aparine L*. In the case of bottom sediment, high variability, depending on the sampling point, was observed for the following studied elements: zinc, manganese, barium, and lead. The differentiation between the zinc content, depending on the sampling point, also was noticeable in the case of plant material. The analogous effect for barium and lead is virtually unnoticeable. For the plant material, the highest variability in concentrations of chemical elements studied was observed for manganese, zinc, and strontium.

The dissimilarity matrix (Fig. 7c) and mean values of the Euclidian distance (Fig. 7d) for the content of the analysed elements in the Bytomka River water also were calculated for five sampling points during 1 year of research. Analysis of the variability of the concentrations of elements over time for each of the measurement points indicated that the highest variability was observed for the fifth sampling point with slightly lower variability for the second and third points, whereas the variability of the concentrations of the analysed elements in the Bytomka River was the smallest for the first sampling point.

**Fig. 8:**
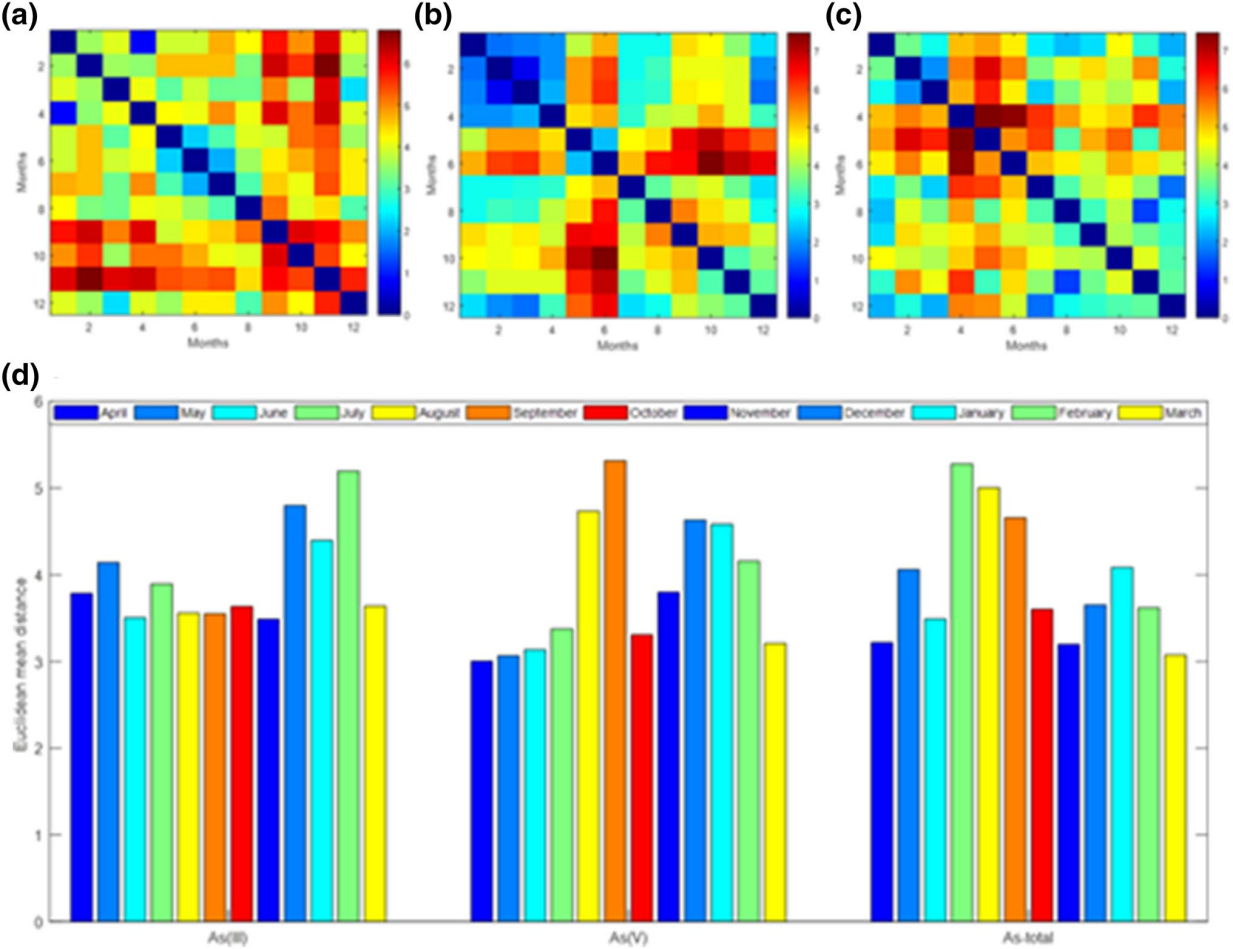
**a** Dissimilarity matrix **D** calculated with the Euclidean distance (the concentration of the As(III) in bottom sediment and water measured in 5 measurement points within 12 months). **b** Dissimilarity matrix **D** calculated with the Euclidean distance (the concentration of the As(V) in bottom sediment and water measured in 5 measurement points within 12 months). **c** Dissimilarity matrix **D** calculated with the Euclidean distance (the concentration of the As-total in bottom sediment and water measured in 5 measurement points within 12 months). **d** Analysis of variation of As(III), As(V), and As-total content in bottom sediment and water for five sampling points during the twelve months of research

The arsenic content in the studied ecosystem was analysed in detail. The second figure (Fig. 8) presents the analysis of the variability in the content of arsenic species and its total content.

The As(III) content shows the highest variability in the winter months (from December to February) and slightly larger fluctuations in May and July. Whereas As(V) also shows significant variability of concentration in winter months as well as in summer months (August and September). The total arsenic content shows the greatest variability in the summer months (from July to September). Also, an increased variability of arsenic concentration in the ecosystem of the Bytomka River is observed in the winter months as well as in the month of May.

#### Cluster Analysis

**Fig. 9:**
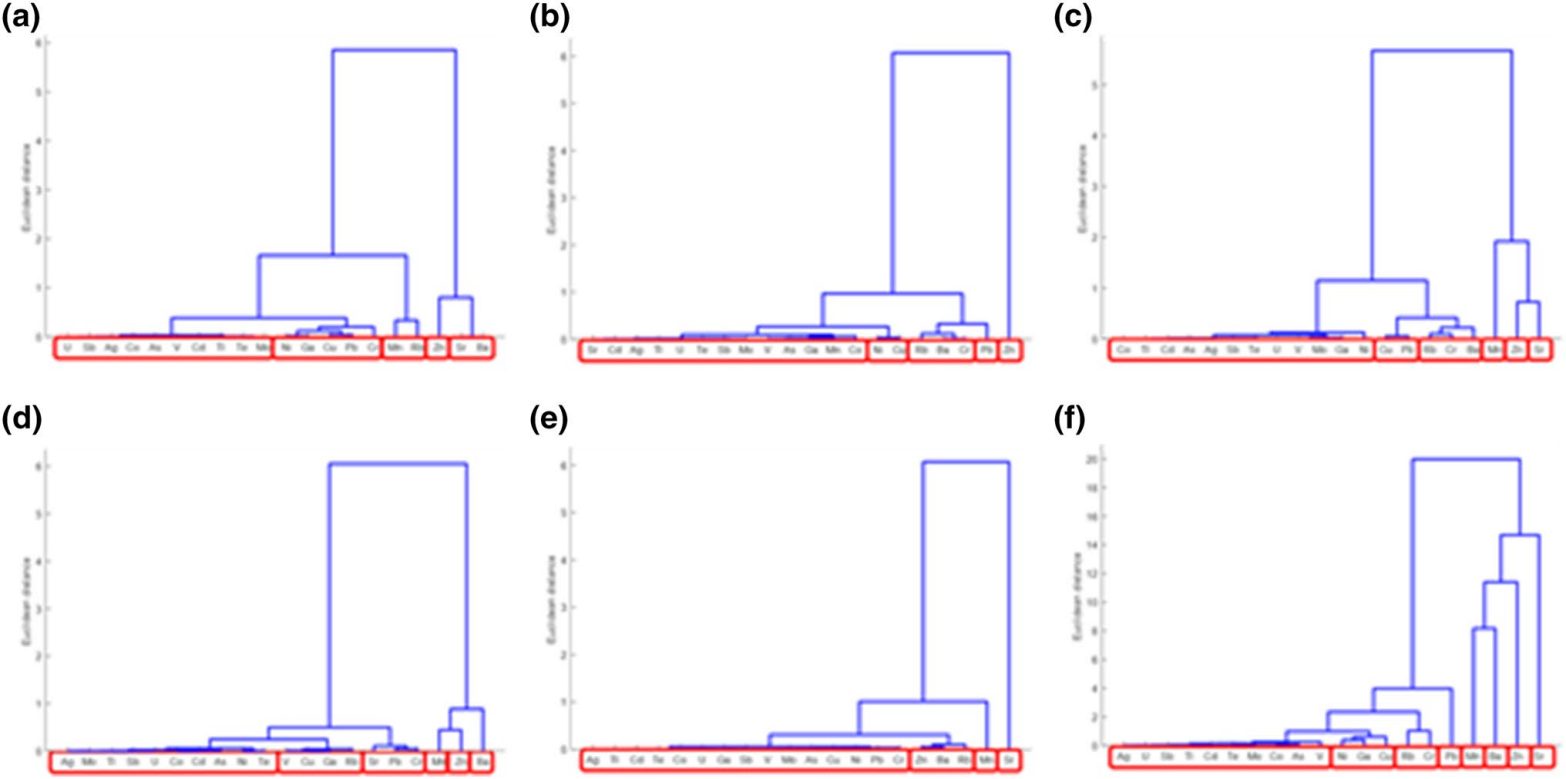
Diagrams of hierarchical clustering showing the similarity in the dynamics of changes in the content of the analyzed chemical elements for: **a** plant material of *Urtica dioica L.*; **b** plant material of *Galium aparine L.*; **c** plant material of *Stuckenia pectinata L*.; **d** bottom sediment; **e** water; **f** ecosystem of the Bytomka River

Dendrograms depict the degree of similarity between the content of chemical elements in the plant material of *Urtica dioica L.* (Fig. 9a), plant material of *Galium aparine L.* (Fig. 9b), plant material of *Stuckenia pectinata L.* (Fig. 9c), bottom sediment (Fig. 9d), water (Fig. 9e), and for ecosystem (plant material, bottom sediment, and water) of the Bytomka River (Fig. 9f).

The hierarchical analysis performed for data obtained from plant material showed the existence of five *Urtica dioica L.* and *Galium aparine L.* or six *Stuckenia pectinata L.* clusters. Also, six clusters occur the analysis of the content of chemical elements in the bottom sediment of the Bytomka River. The data obtained as a result of the analysis of concentrations of the studied chemical elements in water show slightly smaller differentiation than in the case of plant material and bottom sediments, as we observe in the diagram (Fig. 9e), through the existence of a smaller number of clusters. A comprehensive analysis of the whole ecosystem of the Bytomka River (plant material, bottom sediment, and water) revealed the existence of eight clusters among the studied elements.

Dendrograms obtained by hierarchical grouping indicate that there is a large group of elements that behave similarly, independent of the material tested (plant material, sediment, or water). This group includes Ag, As, Cd, Co, Mo, Sb, U, Te, and Tl. On the other hand, elements that in most cases form one-element clusters exhibit the highest degree of bias in relation to other analysed chemical elements; these include Mn, Sr, and Zn. In the case of barium and lead, there also are one-element clusters, which we observe in the diagram illustrating the relationships occurring for the entire Bytomka River ecosystem. For the barium, the analogous situation is observed on the dendrogram obtained on the basis of bottom sediment analysis results and in the case of lead on the basis of results obtained from the analysis of plant material *Galium aparine L*. Also, Fig. 9f indicates that the dynamics of the presence of nickel in the ecosystem is similar to the dynamics of the occurrence of copper. The confirmation of these observations are data obtained for plant material of *Urtica dioica L.* and *Galium aparine L*. Similar conclusions can be drawn by analysing chromium and rubidium contents in the examined environmental material. Both the dendrogram obtained for the whole ecosystem and the dendrograms obtained for the plant material indicate a similar dynamic of changes in chromium and rubidium concentrations.

#### Principal Component Analysis

The initial data set has been divided into three parts to better visualize the relationship between the contents of the studied chemical elements in the environmental material. The first set of data contains concentrations of chemical elements in bottom sediment, water, and plant material *Urtica dioica L*. In the second and third datasets, *Urtica dioica L.* data were replaced with data for *Galium aparine L.* and *Stuckenia pectinata L.*, respectively.

**Fig. 10:**
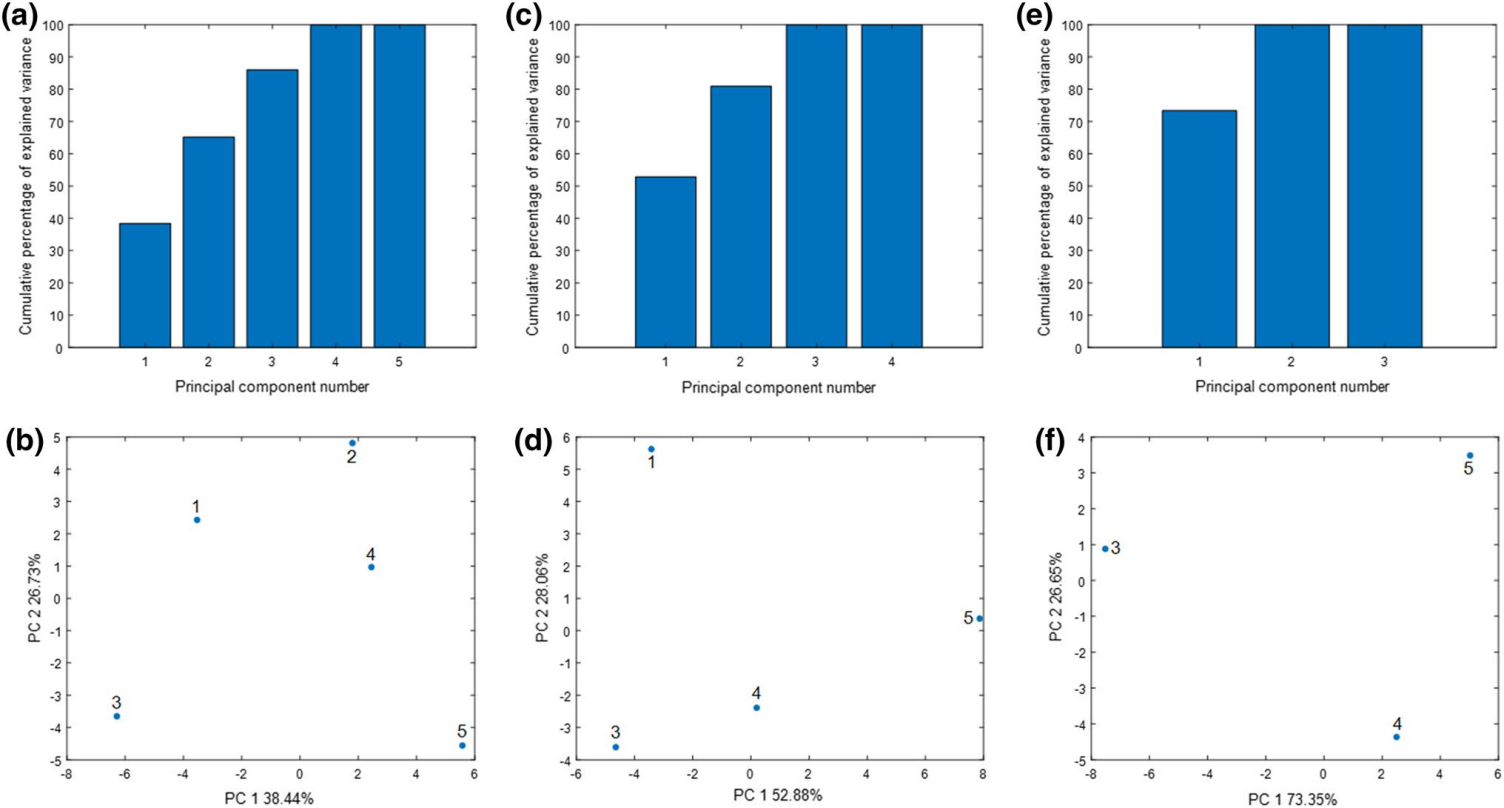
Cumulative percentage of explained variance by successive principal components for data covering: **a** bottom sediment, water, and *Urtica dioica L.*; **c** bottom sediment, water, and *Galium aparine L.*; **e** bottom sediment, water, and *Stuckenia pectinata L*. Projection of objects onto the plane PC1, PC2 for data covering: **b** bottom sediment, water, and *Urtica dioica L.*; **d** bottom sediment, water, and *Galium aparine L.*; **f** bottom sediment, water, and *Stuckenia pectinata L*. The numbers in the figures correspond to the sampling points

The PCA calculations performed for the first dataset showed that the first two principal components explain over 65% of the data variance (PC1—38.44%, PC2—26.73%) (Fig. 10a). For the second data set, the corresponding values are 80% (PC1—52.88%, PC2—28.06%) (Fig. 10c). For the third data set, the first two principal components explain 100% of the data variance (PC1—73.35%, PC2—26.65%) (Fig. 10e). The analyses of the PC1 and PC2 (Fig. 10b, d, and f) projections demonstrate that objects belonging to the sampling points number 3 and 5 had the biggest contribution to PC1 for all data sets, which proves the conclusions from the dissimilarity matrix analysis.

The largest contribution to the second factor is made by the samples taken in the second and fifth points for the first data set (Fig. 10b). For the data set number 2, the largest contributions to the second factor are made by samples collected at the first and third points (Fig. 10d). Data set number 3 is characterized by the largest contribution to PC2 of samples from the fourth and fifth sampling points (Fig. 10f).

**Fig. 11:**
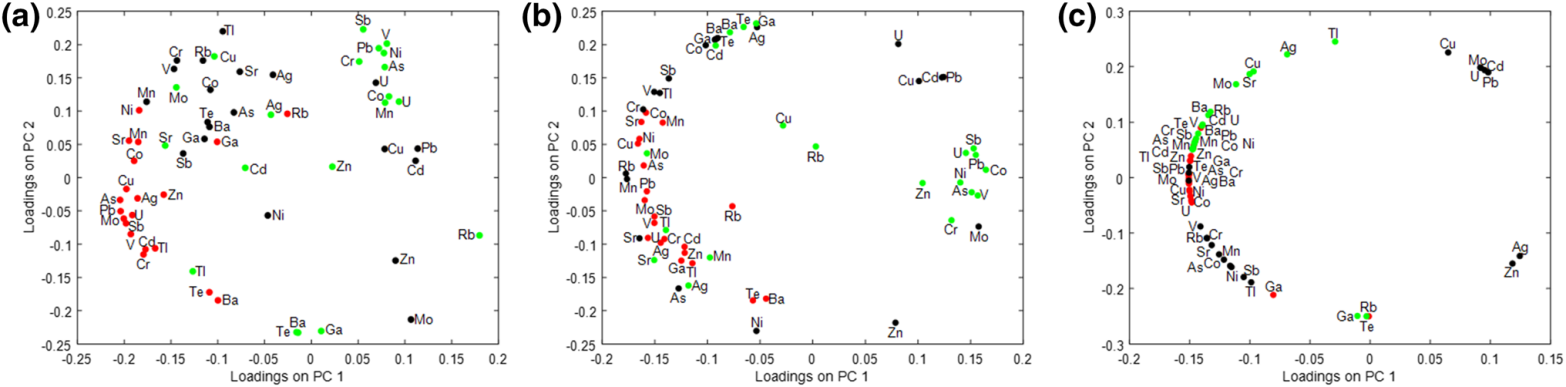
Projection of loadings onto the plane PC1, PC2 for: **a** bottom sediment, water and *Urtica dioica L.*; **b** bottom sediment, water and *Galium aparine L.*; **c** bottom sediment, water and *Stuckenia pectinata L*. Colors are marked with loadings determined for the content of the analyzed chemical elements in the studied environmental materials: red—bottom sediment; black—water; green—plant material

The correlations between the analysed parameters (concentration of elements in the examined environmental material) are shown in the diagrams of loadings (Fig. 11). The dependencies determined by the PCA method were as previously calculated for the three data systems. The first set of data allows one to analyse the correlation dependence of the content of analysed chemical elements in bottom sediment, water, and plant material *Urtica dioica L*. Subsequent data sets allowed for the determination of the correlation dependence between the concentrations of the examined elements in bottom sediment, water, and plant material, *Galium aparine L.* (second set of data) and *Stuckenia pectinata L.* (third set of data).

For bottom sediment collected at five sampling points, a strong positive correlation between the content of arsenic, zinc, copper, silver, lead, molybdenum, uranium, antimony, and vanadium was observed. Another group of chemical elements present in the bottom sediment whose contents are strongly correlated positively is Cd, Cr, and Tl. Also, concentrations of manganese, strontium, and cobalt in the bottom sediment show a strong positive correlation. An analogous relationship also is found between barium and tellurium concentration in the bottom sediment and between the concentration of gallium and nickel.

For water, the strongest positive correlations are observed for copper, cadmium, and lead concentrations. The content of As, Ag, Ba, Co, Cr, Ga, Mn, Rb, Sr, Te, Tl, and V also have strong positive correlations. In the plant material *Urtica dioica L.*, there is a strong positive correlation between the contents of the following elements: As, Co, Cr, Mn, Ni, Pb, Sb, U, and V. Also, a positive correlation is observed for barium, gallium, and tellurium. However, there is a negative correlation between the groups indicated above.

The correlation relationships occurring between the contents of the studied elements in bottom sediment, water, and plant material are extremely important to assess the dynamics of changes in the concentrations of chemical elements in the ecosystem. The research indicated that concentrations of Ga and Mn in the bottom sediment and water are positively correlated with each other, whereas there also is a group of elements (exactly their content in the bottom sediment and in water), which shows a negative correlation. This group includes copper, cadmium, lead, and uranium. The analysis of the content of the studied chemical elements in the plant material *Urtica dioica L.* indicates that there is a strong positive correlation between the concentrations of strontium in the plant material and bottom sediment. However, for silver, molybdenum, and uranium, there is a positive correlation between the contents in water and plant material. For the other chemical elements considered in this paper, a weak correlation or lack thereof was observed.

The analysis of the content of the tested chemicals in the plant material *Galium aparine L.* showed a strong negative correlation relationship with the content of the following chemical elements in the bottom sediment: As, Ni, Pb, Sb, and U. The concentrations of Ba, Sr, and Te in water strongly correlate positively with the concentrations of these chemical elements in the plant material *Galium aparine L*. An interesting observation is the strong negative correlation between the content of chromium in water and the content of this element in the plant material *Galium aparine L*.

In the case of *Stuckenia pectinata L.*, there is a different situation than for *Urtica dioica L.* and *Galium aparine L*. The content of many of the elements in the plant material *Galium aparine L*. strongly correlate positively with the content of these elements in the bottom sediment. This group includes As, Ba, Cd, Mn, Te, and Zn. However, in the case of the content of the elements studied in water, a positive correlation is observed for lead and antimony. It is worth noting that in the case of silver content in the water of the Bytomka River, as well as the content in the plant material *Galium aparine L*., there is a strong negative correlation that is not observed for other chemical elements.

## Conclusions

The Bytomka River is subject to a strong urban-industrial anthropopression. It is heavily contaminated with heavy metals and metalloids (class IV in the LAWA classification). The Pollution Load Index (PLI) and geoaccumulation Index (*I*_geo_) values clearly indicate significant pollution of the examined ecosystem with heavy metals. Increased water and bottom sediment pollution considerably influences the content of metals and metalloids in coastal plants, such as *Stuckenia pectinata L.*, *Galium aparine L.,* and *Urtica dioica L.* High arsenic content in the bottom sediment and in the river water over which the plants grow significantly increases the concentration of this metalloid in plants. Subjected to strong anthropopressure, plants try to protect themselves against the harmful effects of high metal and metalloid concentrations, among others, by methylation processes. Hence, the results of our investigations show the presence of methyl derivatives, such as MMA and DMA in plant samples.

Among the studied plants growing on the contaminated area, *Stuckenia pectinata L.* demonstrated the highest arsenic, lead, and zinc concentration. It most easily accumulated the discussed element from the substrate. In addition, *Urtica dioica L.* was characterized by a high content of metals and metalloids. Both *Urtica dioica L.* and *Stuckenia pectinata L.* are plant species that can help to rebuild areas exposed to metal and metalloid contamination by storing these contaminants. Both these plants species naturally grow on riverbanks around the world, thereby cleansing river ecosystems of anthropogenic pollution.

The use of chemometric methods as well as PLI and *I*_geo_ coefficients in the analysis of environmental data allowed for a comprehensive description of the ecosystem contaminated with heavy metals. In this work, it was particularly valuable to show the dynamics of heavy metals in an ecosystem (water, bottom sediment, and coastal vegetation) subjected to strong anthropogenic pressure.

The values of PLI and *I*_geo_ coefficients determined for five sampling points confirmed that the Bytomka River ecosystem is polluted with heavy metals to a significant degree. The highest concentrations of the heavy metals tested were recorded for the third sampling point. Similarity analysis showed the largest range of variability of concentrations of the analysed heavy metals in the ecosystem studied for the fifth sampling point, especially during the winter months (December, January, February). In particular, the arsenic content and its species in the ecosystem of the Bytomka River were analysed in detail. The research conducted showed significant differences in the dynamics of As(III) and As(V) depending on the season and place of sampling. For As(III), the highest variability of concentration occurs in the winter months (December, January, February). While for As(V), the highest variability of concentration is observed in the summer months (August, September). The cluster analysis and principal component analysis allowed us to examine the mutual relationships between the contents of the chemical species present in water, bottom sediments, and coastal vegetation. This is very important information, which enables an assessment of the dynamics of the spread of pollutants, such as heavy metals in the natural environment.

## Data Availability

All chemometrics calculations were performed on a PC equipped with an Intel^®^ Core™ i7-Q740 and a 1.73 GHZ processor with 16 GB RAM using MATLAB (version R2018b) running under Windows 10 Professional (64-bit system).
